# Panoramic Uncertainty in Vertical Perception

**DOI:** 10.3389/fnint.2021.738768

**Published:** 2021-11-17

**Authors:** Janny C. Stapel, W. Pieter Medendorp

**Affiliations:** ^1^Donders Institute for Brain, Cognition, and Behaviour, Radboud University, Nijmegen, Netherlands; ^2^Uppsala Child and Babylab, Department of Psychology, Uppsala University, Uppsala, Sweden

**Keywords:** multisensory integration (MSI), subjective visual vertical (SVV), Bayesian, vision, vestibular, rod-and-frame

## Abstract

Judgments of the orientation of a visual line with respect to earth vertical are affected by panoramic visual cues. This is illustrated by the rod-and-frame effect (RFE), the finding that the perceived orientation of a luminous rod is biased by the orientation of a surrounding squared frame. In this study, we tested how the uncertainty of frame orientation affects the RFE by asking upright or tilted participants to psychometrically judge the orientation of a briefly flashed rod contained within either a circular frame, a squared frame, or either of two intermediate frame forms, called squircles, presented in various orientations. Results showed a cyclical modulation of frame-induced bias across the range of the square and squircular frame orientations. The magnitude of this bias increased with increasing squaredness of the frame, as if the more unequivocal the orientation cues of the frame, the larger the reliance on them for rod orientation judgments. These findings are explained with a Bayesian optimal integration model in which participants flexibly weigh visual panoramic cues, depending on their orientation reliability, and non-visual cues in the perception of vertical.

## Introduction

**FIGURE 1:**
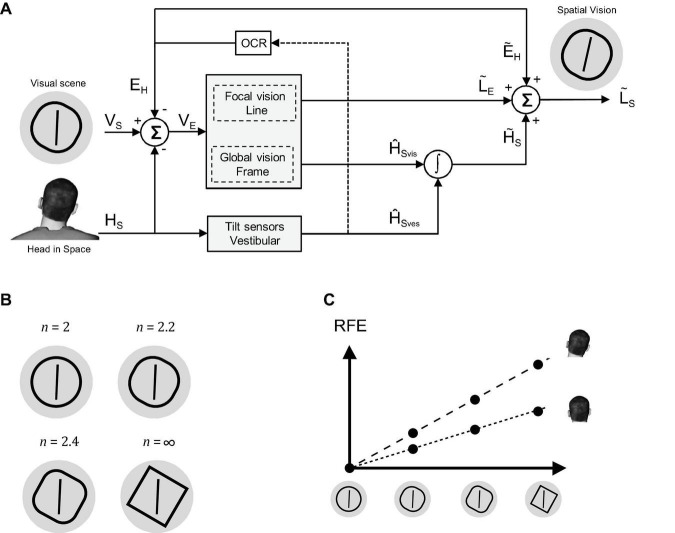
**(A)** A schematic of the Bayesian model. **(B)** The four frame forms used: circle (*n* = 2), squircle 2 (*n* = 2.2), squircle 1 (*n* = 2.4), and square (*n* = ∞). **(C)** Hypothesized relation between frame form, head orientation, and magnitude of the rod-and-frame effect.

Many of our daily activities, such as walking, standing, or gaze control, rely on estimates of head and body orientation in space. These estimates are inferred not only from sensory inputs, such as visual and vestibular cues but are also based on motor feedback and prior expectations. As a measure of spatial orientation, experimentalists often assess the percept of vertical, i.e., the perceived orientation of a visual line relative to gravity (${\overset{∽}{L}}_{G}$), which can computationally (Figure 1A) be inferred by combining the orientation estimates of the head (${\overset{∽}{H}}_{G}$), eye-in-head (${\overset{∽}{E}}_{H}$), and line-on-retina (${\overset{∽}{L}}_{E}$), according to ${\overset{∽}{L}}_{G}={\overset{∽}{H}}_{G}+{\overset{∽}{E}}_{H}+{\overset{∽}{L}}_{E}.$ How do visual cues contribute to the perception of vertical? Rich visual scenes typically contain various panoramic cues, such as houses, trees, or the horizon. These cues unambiguously indicate which direction is up and hence can provide the brain with information about gravity direction (van der Schaaf and van Hateren, 1996; Coppola et al., 1998; Girshick et al., 2011; Pomante et al., 2021). More impoverished visual scenes, lacking clear panoramic cues, also affect the percept of vertical (Ebenholtz and Callan, 1980; Li and Matin, 2005a). For example, the perceived orientation of an earth-vertical line is biased when surrounded by a tilted squared frame (Witkin and Asch, 1948; Alberts et al., 2016; Niehof et al., 2019), an effect known as the rod-and-frame effect (RFE; Witkin and Asch, 1948). The magnitude of this RFE cyclically changes as the frame rotates across a 90° range (Wenderoth, 1973; Alberts et al., 2016). In fact, even a single peripheral line could induce such a bias, with the same 90° periodicity (Vingerhoets et al., 2009). Matin and Li (1995) explained the RFE as an indirect contribution of the visual frame to the internal estimate of head orientation. In turn, this visual signal, in combination with vestibular and other non-visual head orientation signals, then affects the perceived orientation of the rod (see Figure 1A). Rules of Bayesian inference dictate that the most precise estimate (estimate with the lowest variance) of head orientation is achieved by integrating the sensory signals and prior expectations according to their reliability (Laurens and Droulez, 2007; MacNeilage et al., 2007; De Vrijer et al., 2008, 2009; Tarnutzer et al., 2009; Clemens et al., 2011; Alberts et al., 2016; Kheradmand and Winnick, 2017; De Winkel et al., 2018, 2021). This means that more reliable information weighs in heavier in the combined head orientation estimate than less reliable information. Alberts et al. (2016), building on the work of Vingerhoets et al. (2009) and Clemens et al. (2011), provided a Bayesian model of the RFE, involving a precision-dependent weighting of vestibular and visual frame signals. They experimentally validated their model by showing that lowering the vestibular reliability by tilting the head (Tarnutzer et al., 2009) increased the RFE and that reducing the visual frame reliability by increasing viewing distance reduced the RFE.

Noteworthy, the manipulation of viewing distance in the study by Alberts et al. (2016) affected the quality of the whole visual scene; the global frame and the local rod. Therefore, it remains elusive whether changes in the retinal size of the rod, the frame, or both caused the alteration of the RFE effect. Recent findings of Pomante et al. (2019) suggest that visual uncertainty of rod orientation is not central. The authors manipulated the orientation reliability of the rod by replacing it with ellipses differing in their polarization from near-circular to strongly polarized. Polarization of the ellipse did not alter the RFE, suggesting that the rod does not function as a head orientation cue (Figure 1A). In the current study, we further tested the role of visual frame reliability in the RFE and its interaction with changes in vestibular reliability.

In contrast to the unambiguous cue to the gravity direction provided by rich visual scenes, a squared visual frame provides less certainty by delivering four ambiguous cues. Heuristically, a circular frame provides no cues to gravity direction, but frames intermediate a square and circle could be expected to differ in the reliability of their implicit cues to the gravity direction. Here, we employed this type of manipulation by contrasting the effects of a square and a circle with two intermediate forms known as *squircles* (see Figure 1B; Weisstein, 2011). A squircle, which is a superellipse with equal length semi-axes, can be specified as:


(1) $${\left|\frac{x-a}{r_{a}}\right|}^{n}+{\left|\frac{y-b}{r_{b}}\right|}^{n}=1$$

where, *r*_*a*_ and *r*_*b*_ represent the length of the semi-axes, and *a* and *b* quantify the offset with respect to the origin. In a squircle, *r*_*a*_ and *r*_*b*_ are equal, and the larger the *n*, the more square-like the form. In the current study, the RFE of a squared frame was compared to a squircular frame with *n* = 2.4, a more circular squircle with *n* = 2.2, and a full circular frame (*n* = 2), under two physical orientations of the head (upright and rightward tilted by 30°).

Reasoning based on the Bayesian model described above, we expected the magnitude of the RFE – the bias – to increase with the squaredness of the frame and with decreasing vestibular reliability (Figure 1C). Likewise, the impact of frame orientation on the response variability was expected to be larger with increasing squaredness and more strongly so when the vestibular reliability was reduced.

## Materials and Methods

### Participants

Data of 12 participants (mean age = 20.5 years, *SD* = 2.9 years, eight women) naïve to the study purposes were included in the final analyses. All included participants finalized two experimental sessions, which took them 2 h per session. Ten additional participants were recruited but excluded from the analyses, for failing to finish the first session (*n* = 3), not returning at the second session (*n* = 3), or not following the task instructions (*n* = 4). All participants had normal or corrected-to-normal vision and no known (history of) neurological disorders. Participants were recruited from a participant database of Radboud University. They provided written informed consent prior to taking part and received either gift vouchers (€20 per 2 h session) or study points for participation.

### Experimental Setup

Participants were seated in a chair that was mounted on a height-adjustable frame. After adjusting the height of the chair and table, the researcher locked the position of the chair. Two custom-made vertical foam-padded headrests were mounted to the frame. The headrests were adjustable in height and position, such that these could be aligned with and gently enclosing the ears of the participant. The headrests stabilized the head either in an upright or in a 30° tilt position.

The participants looked through a tube (length: 70 cm, diameter: 31.5 cm) in front of them toward an OLED TV screen (LG 55EA8809, 123 × 69 cm, 1,920 × 1,080 pixels, refresh rate 60 Hz) in a darkened laboratory. The advantage of an OLED screen is that pixels set to black do not emit light. A tunnel of cloth connected the head of the participant with the front of the tube. Both cloth and tube were used to prevent potential remaining external light to reach the eyes of the participants. Participants had to indicate with a handheld button-box whether the rod was rotated clockwise (CW) or counterclockwise (CCW) relative to the earth vertical.

### Experimental Procedure

The participants performed a rod-and-frame task, in which they judged whether a rod presented against a background of a frame was rotated CW or CCW with respect to the gravitational vertical. Each trial started with a gray frame (square: 15 × 15 cm, circle and squircles: diameter of 15 cm, 12.2° visual angle) presented on a black background. After 200 ms, a gray rod (length: 12 cm, 9.8° visual angle, width: 1 px) was presented in the center of the frame for 33 ms, and then the rod was removed again. The frame remained on the screen until the participant had responded by pressing one of the two available buttons. Between trials, the screen was black for 400 ms.

The orientation of the rod and the orientation of the frame were varied independently. The rod’s orientation was randomly selected from a set of nine rod orientations centered around the gravitational vertical (−7°,−4°,−2°,−1°, 0°, 1°, 2°, 4°, or 7°), and the frame was displayed in an orientation randomly chosen from a set of 15 angles between −35° and 35°, in steps of 5°. Four different frame forms were used: a square, a circle, and two squircles (see Figure 1B). Except for the circle, all possible combinations of frame form, frame orientation, and rod orientation were used. Because a circle has no orientation, only the rod orientation was varied in the circle condition. This led to 414 unique trials, together constituting a sequence. The experiment was split into two sessions and each session consisted of 10 trial sequences. A session thus consisted of 4,140 trials and typically took about 2 h including breaks. The trial order was randomized within each sequence. Head orientation was either held upright or tilted 30° to the right within a session. The order of head orientation conditions was counterbalanced across participants. Both sessions started with 10 practice trials. The performance of participants was monitored during practice trials, and, if needed, another practice round was included.

### Data Analyses

The data analyses, which will be explained in more detail below, included a number of steps. First, psychometric curves were fitted to the data, and a summary statistic describing the goodness-of-fit (the Bayesian information criterion, BIC) was calculated. The psychometric fits provided a model-free benchmark for comparison with the Bayesian model. Next, the Bayesian model was fitted to the data and the same summary statistic was obtained. Subsequently, the Bayesian model was validated and evaluated by means of a bootstrapping procedure and a parameter recovery analysis. Finally, the goodness-of-fit of the Bayesian model was compared to the psychometric model-free benchmark.

### Model-Free Benchmark

Clockwise frame and rod orientations were defined positively. Per participant, session, frame form, and frame orientation, a cumulative Gaussian was fit to the proportion of CW responses as a function of rod orientation (Wichmann and Hill, 2001):


(2) $$P\,\left(x\right)=\lambda +\left(1-2\,\lambda \right)\,\frac{1}{\sigma \,\sqrt{2\,\pi }}\,\int_{-\infty }^{x}e^{-{\left(y-\mu \right)}^{2}\,/\,2\,\sigma^{2}}\,d\,y$$

where *x* represents the rod orientation in space and λ the lapse rate, accounting for individual stimulus-independent errors. The mean μ and the SD σ of the Gaussian account for the perceived orientation of gravity of participants [i.e., the systematic bias or point-of-subjective equality (PSE)] and response variability, respectively. A Matlab search routine called “fminsearch” was used to find the fit that maximized the likelihood estimation, through searching for the minimum of the negative log likelihood.

### Model Fitting

A Bayesian optimal integration model was fitted on the responses of the participants. This model has been described in full detail in a previous paper (Alberts et al., 2016). In short, the model describes that how a line-on-retina estimate can result in a line-in-space estimate via a few steps. First, the line-on-eye estimate is combined with the eye-in-head estimate, producing a line-relative-to-head estimate. This line-relative-to-head estimate can then be combined with the head-in-space estimate to result in a line-in-space estimate. According to the model, the head-in-space estimate results from optimally integrating (extra)vestibular cues, the visual contextual cues, and the prior on head orientation, following:


(3) $$P\,\left({\overset{∽}{H}}_{s}|{\hat{H}}_{s},{\hat{\theta }}_{R}\right)=P\,\left({\hat{H}}_{s}|H_{s}\right)\cdot P\,\left({\hat{\theta }}_{R}|H_{s}\right)\cdot P\,\left(H_{s}\right)$$

where *P* ($\hat{H}$*_*s*_* | *H*_*s*_) is the vestibular likelihood, *P* ($\hat{\theta }$ *_*R*_* | *H*_*s*_) is the contextual likelihood, and *P* (*H*_*s*_) is the prior head orientation. The head-in-space estimate with the highest probability given the sensory evidence is utilized: the *maximum a posteriori* (MAP). The vestibular likelihood is based on the (extra)vestibular cues, which are assumed to be veridical but contaminated with noise. The vestibular noise parameter is operationalized as an offset (β*_*HS*_*) plus a noise component that scales linearly with the tilt angle of the head relative to the upright position (α*_*HS*_*). The prior on head orientation relative to the earth vertical was modeled as a Gaussian centered at 0° (upright) with SD σ*_*HP*_*. The contextual likelihood is described in the model as a function that strongly relies on the sensory inputs from the cardinal directions of the frame. The sensory input is expressed here in retinal coordinates and we accounted for the uncompensated ocular counterroll in the head-tilted condition by including a parameter *A*_*OCR*_, which cannot be established empirically. To remove ambiguity in the fitting of the present data, we fixed the *A*_*OCR*_ at 14.6 based on previous findings (Clemens et al., 2011; see Alberts et al., 2016 for mathematical details). The contextual probability distribution is modeled as the normalized sum of four von Mises distributions, with one von Mises distribution peaking at the veridical frame orientation, and the others peaking at 90° intervals. As previous work demonstrated that vertical perception more strongly relies on the (near) vertical frame lines than on the (near) horizontal lines (Alberts et al., 2016), the variance in the vertical direction (σ*^2^_*ver*_*) was allowed to vary independently from the variance in the horizontal direction (σ*^2^_*hor*_*). Additionally, changes in frame orientation will also change the dependence on the cardinal axes, with equal importance for the horizontal and vertical at a frame orientation of 45° and a lessening importance of the horizontal for more upright frames. The change in dependence on the different cardinal axes was modeled as a free parameter *τ*.

The described model had seven free parameters (σ*_*HP*_*, α*_*HS*_*, β*_*HS*_*, σ*_*ver*_*, σ*_*hor*_*, *τ*, and λ), with λ denoting the lapse rate. All data were first symmetrized per participant and condition (as in Clemens et al., 2011; Alberts et al., 2016), based on the responses of the participants to the head upright square frame condition. Then, the model was fit per participant on the data of the square frame fitting both vestibular conditions simultaneously. Subsequently, the obtained parameter values were fixed and used to fit the data of the squircles, with only one free parameter, namely, a gain factor. The gain factor, *g*_1_ for squircle 1 and *g*_2_ for squircle 2, scaled the variability in visual context probability distribution such that a gain of one implied that σ*_*ver*_* and σ*_*hor*_* in the squircle condition were equal to those in the square condition, whereas, a gain factor larger than one implied increased variability in comparison to the squared frame.

Model fitting was performed in Matlab 2015b (Mathworks) using the function “fmincon” to minimize the log likelihood of the data given the parameter values. Random initial parameter values were used, and the routine was repeated five times to ensure a global rather than a local minimum would be found.

### Model Evaluation

Hundred bootstrap runs were performed to obtain the SD of the fitted parameter values. Per run, 1,350 stimuli (15 frame orientations × 9 rod orientations × 10 repetitions) and accompanying responses were randomly sampled with replacement from the original data.

To validate our fitting procedure, we performed a parameter recovery analysis to ensure that they can be inferred well given our experimental design and analysis pipeline (see Perdreau et al., 2019 for further details; here, bootstrapped parameter values were used). We determined to what extent the recovered parameter values could be predicted from the initial parameter values by means of linear regression analyses. The variance explained by the regression (*R*^2^) was taken as an indicator of the validity of the Bayesian fitting procedure.

The model was furthermore evaluated by comparing the bias and variability obtained from fitting the cumulative Gaussian with the bias and variability data obtained through forwarding modeling using the parameter values resulting from the bootstrapping. As an indication of the quality of the model fits, the BIC (Schwarz, 1978; Raftery, 1995) was computed for both the full model fits and the psychometric fits (as a descriptive account of the data). The BIC for the psychometric fits included both head orientations, three frame forms, and all 15 frame orientations, resulting in 186 free parameters, to allow a comparison with the BIC values of the full model, which had seven free parameters for fitting the data of the square, and one free parameter for fitting the data of each squircle (hence nine free parameters in total). The BIC trades off the likelihood of a model given the data and the number of free parameters, following:


(4) $$B\,I\,C=k\,\log\left(n\right)-2\,\text{log}\,\left(\hat{L}\right)$$

where *k* is the number of free parameters, *n* is the number of observations, and $\hat{L}$ is the maximum likelihood of the data given the model. The BIC is useful for comparing models which differ in the number of free parameters. Lower BIC values indicate a better fit. We also subjected the gain factors of the three frame forms (all except the circle) to a statistical comparison.

## Results

**FIGURE 2:**
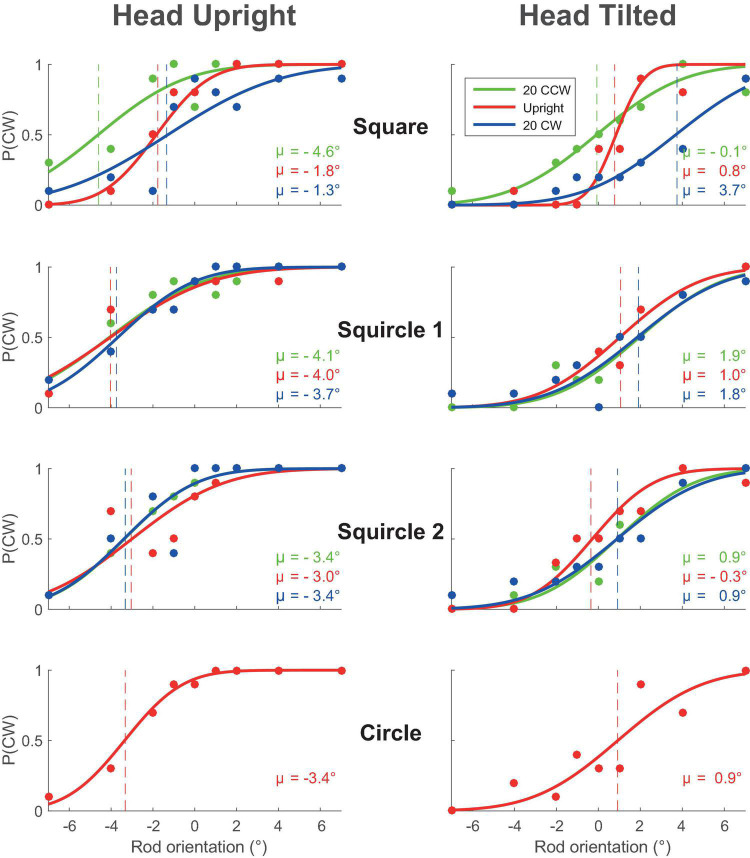
The lines represent the psychometric curves that best fit the data of one of the participants. The raw data points (proportion of CW responses) are shown as circles. Panels on the left correspond to the head upright condition, to the right to the head tilted condition. The panels from top to bottom correspond to the used frame forms: square, squircle 1, squircle 2, and circle. The different colors represent different frame orientations (green, 20 degrees CCW; red, 0 degrees; blue, 20 degrees CW). CW, rotated clockwise; CCW, counterclockwise.

Figure 2 shows the data of a single participant as the proportion of CW responses at each rod orientation for the squared frame and the two squircles in three exemplar frame orientations: 20°CCW, 0° and 20°CW, and the circular frame, during the head upright (left) and head tilted (right) condition. We fitted psychometric curves based on the obtained bias and variability of the responses (see section “Materials and Methods”). In the first row, depicting data for a squared frame, three central characteristics of the RFE can be observed. First, the bias shifts with the orientation of the frame: the dotted red line, representing the point of subjective equality for the upright frame (i.e., the orientation of the rod for which an equal number of CW and CCW responses was given) is located to the right of the dotted green line, which represents the PSE for the 20° CCW-oriented frame, and to the left of the blue line, which represents the 20° CW frame. Second, the red curve is steeper than the blue and green curve, which indicates that the rod orientation estimate is more precise when the frame is upright rather than tilted. A third characteristic of the RFE can be found when comparing the upper left and the upper right panel, namely, that the PSE shifts with head tilt.

The effect of frame form on the RFE follows from the comparison among the different rows of panels. As shown, the shift in the PSE due to the frame orientation appears to be more substantial for the squared frame than for the two squircular frames, conform our expectation that the RFE is stronger for more square-like forms. Furthermore, the difference in steepness between the colored lines seems more pronounced in the square frame condition than in the squircular frame conditions. This is again in line with the expectation that the effect of the frame is stronger for squared compared to squircular frames.

**FIGURE 3:**
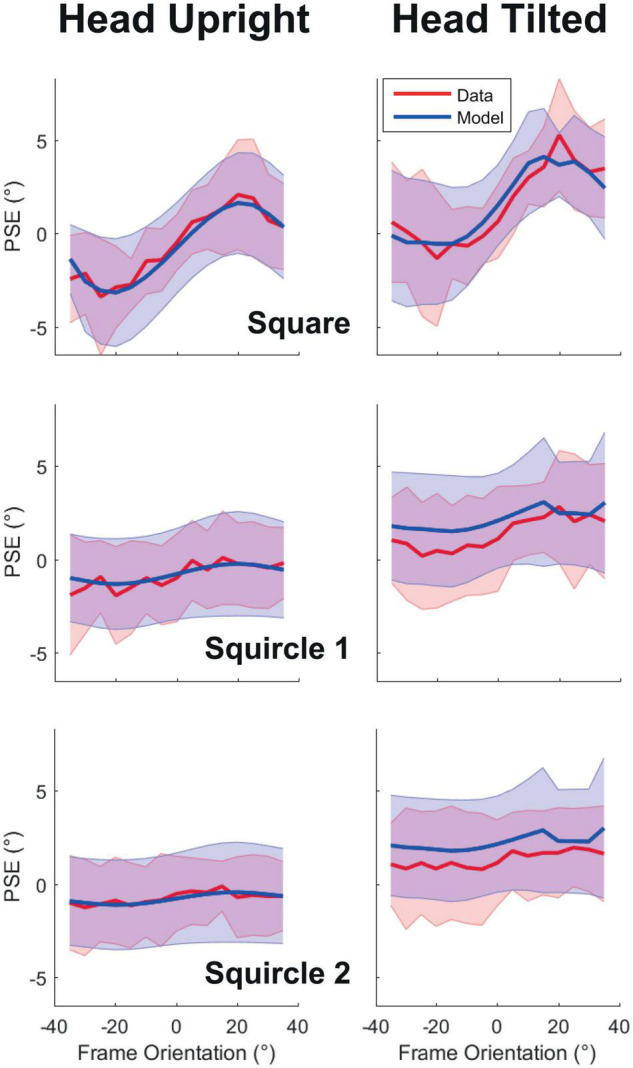
Point-of-subjective equality as a function of frame orientation. Panels on the left correspond to the head upright condition, to the right to the head tilted condition. The panels from top to bottom correspond to the used frame forms: square, squircle 1, and squircle 2. Grand averages are plotted as lines, standard deviation over participants as shaded regions. In red the observed data, in blue the results of the best-fit model. PSE, point-of-subjective equality.

Figure 3 illustrates the observed and modeled PSE of the square-like frames as a function of frame orientation, as an average across participants. If the PSE is zero, the rod orientation judgments are unbiased, whereas, when the PSE is systematically off from zero, there is a bias. With a squared frame (upper panels), a clear cyclical pattern is visible in the measured bias, which is the classic observation about the RFE, the bias is negative for CCW-oriented frames and positive for CW frames. Furthermore, the modulation of the systematic error appears stronger when the head is tilted (panels on the right) compared to when the head was upright (panels on the left). The cyclical pattern seen with squared frames is reduced for squircle 1, and even more strongly so for squircle 2. In other words, the RFE appears to reduce with increasing roundness of the frame form, as predicted. The plots further show that tilting the head led to a larger bias in rod orientation judgments in the presence of the squircular frames.

Figure 3 further shows a relatively close overlap between the model and the data, suggesting that the model performed well in capturing the biases of the participants. The model fitted best for the systematic data from the square frame (mean BIC = 2,539), followed by squircle 1 (mean BIC = 2,903) and squircle 2 (mean BIC = 2,913). Moreover, the full model provided a better fit with the data than a purely descriptive account of the data (i.e., by fitting separate psychometric curves to the data, see Table 1).

**TABLE 1:** Delta BIC values (BIC_psy_ – BIC_full_).

Participant	Psychometric fits – Full model
P1	1,048
P2	664
P3	985
P4	−662
P5	720
P6	1,272
P7	1,191
P8	−51
P9	1,265
P10	109
P11	1,156
P12	1,123
Overall	14,100

**FIGURE 4:**
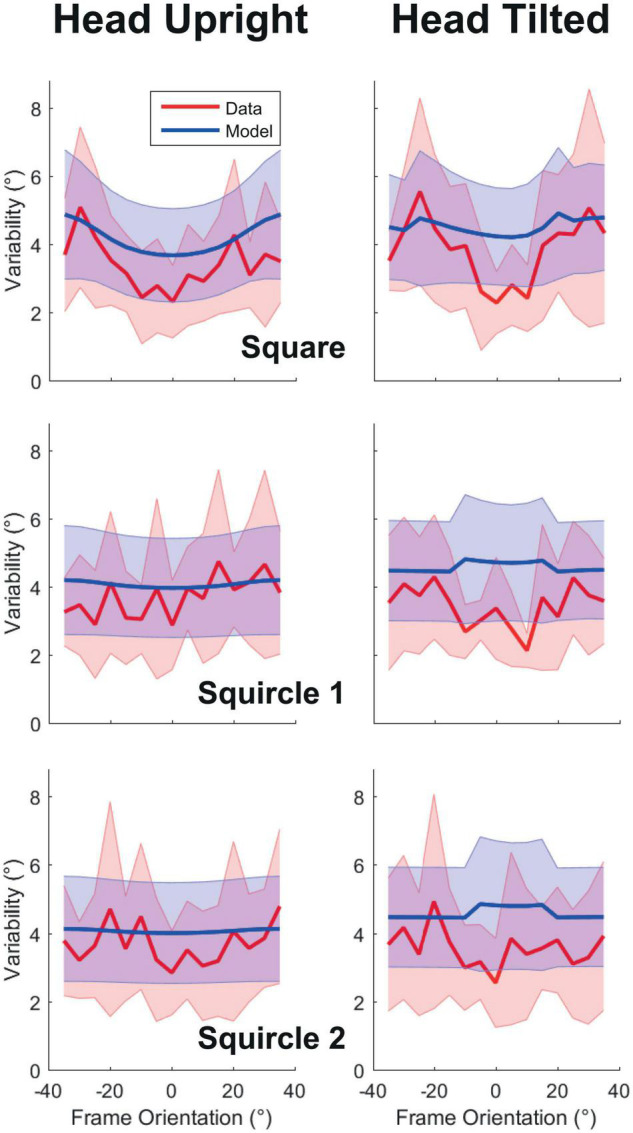
Response variability as a function of frame orientation. Panels on the left correspond to the head upright condition, to the right to the head tilted condition. The panels from top to bottom correspond to the used frame forms: square, squircle 1, and squircle 2. Grand averages are plotted as lines, standard deviation across participants as shaded regions. In red are the observations, in blue, the results of the best-fit model.

Figure 4 displays the observed and modeled variability for each frame form as a function of frame orientation. For the squared frame, a V-shaped pattern can be observed in the data with the lowest variability around upright, closely resembling earlier findings (Alberts et al., 2016; Niehof et al., 2019). Head tilt appeared to lead to a stronger modulation of variability by frame form. This was expected: an ideal observer should rely more strongly on frame orientation because the vestibular derived orientation cues are noisier when the head is tilted. Furthermore, as predicted, the impact of frame orientation decreases dramatically with increasing roundness of the frames, and the pattern in the variability data is flatter for squircles 1 and 2 than for the square. The model does not perfectly capture the trends in the observed response variability. The reason is that the model overestimates variance to allow some wiggle room for fitting the systematic error in the responses.

Table 2 lists the bootstrapped derived parameter values (± SD) for each participant. The model was first fitted on the data of the squared frame, fitting both head tilt conditions simultaneously (see section “Materials and Methods”). While there is some variability across participants, on average the parameters (σ*_*HP*_*, α*_*HS*_*, β*_*HS*_*, σ*_*ver*_*, σ*_*hor*_*, and *τ)* match fairly well with earlier reports (Clemens et al., 2011; Alberts et al., 2016). With these parameters established, we fitted the gain factors, *g*_1_ for squircle 1 and *g*_2_ for squircle 2, which scaled the variances in visual context probability distribution to that of the squared frame. While there was some variability across participants, the gain factor of squircle 1 was significantly larger than 1 (*M* = 32.6, *SD* = 23.1; *t*(11) = 4.745, *p* = 0.001; Cohen’s *d* = 1.4), indicating that the variance of the visual context was higher for squircle 1 compared to the squared frame. In other words, rounding the square reduced the precision of the panoramic cue, as expected. Similarly, the gain factor was higher for squircle 2 compared to squircle 1 in 9 of the 12 participants, suggesting that rounding the frame has a parametric impact on panoramic cue precision.

**TABLE 2:** Parameter values of the model, including a measure of how well the parameter values could be recovered (*R*^2^).

Participant	σ_*HP*_(°)	α_*HS*_(°/°)	β_*HS*_(°)	σ_*ver*_(°)	σ_*hor*_(°)	τ	λ	*g*_1_	*g*_2_
P1	19.2 ± 2.9	0.31 ± 0.04	5.6 ± 1.5	4.9 ± 1.1	159 ± 41	0.75 ± 0.05	0.06 ± 0.05	1.6 ± 0.0	2.0 ± 0.0
P2	3.9 ± 0.3	0.00 ± 0.00	3.4 ± 0.3	5.3 ± 2.1	159 ± 48	0.76 ± 0.13	0.04 ± 0.02	47.8 ± 19.3	57.4 ± 5.3
P3	8.1 ± 0.8	0.00 ± 0.00	6.2 ± 0.8	107.4 ± 71.2	33 ± 52	0.75 ± 0.38	0.07 ± 0.05	53.8 ± 13.4	14.9 ± 12.6
P4	4.4 ± 0.4	0.05 ± 0.01	2.5 ± 0.4	2.8 ± 1.1	127 ± 57	0.71 ± 0.03	0.11 ± 0.02	45.1 ± 25.0	47.4 ± 24.0
P5	7.4 ± 0.6	0.01 ± 0.01	4.1 ± 0.4	7.8 ± 1.6	92 ± 63	0.84 ± 0.10	0.04 ± 0.02	8.8 ± 11.8	12.6 ± 14.3
P6	16.8 ± 2.4	0.00 ± 0.00	7.8 ± 0.9	22.0 ± 6.1	124 ± 66	0.54 ± 0.37	0.14 ± 0.04	11.4 ± 14.5	13.8 ± 12.3
P7	13.4 ± 3.6	0.07 ± 0.03	5.1 ± 0.7	8.7 ± 0.8	97 ± 58	0.76 ± 0.15	0.12 ± 0.05	59.2 ± 2.3	55.4 ± 5.7
P8	6.2 ± 0.5	0.04 ± 0.02	3.7 ± 0.6	6.6 ± 2.0	119 ± 75	0.75 ± 0.15	0.15 ± 0.01	45.4 ± 20.2	50.9 ± 17.1
P9	7.8 ± 0.5	0.01 ± 0.01	3.1 ± 0.2	7.0 ± 0.9	19 ± 28	0.97 ± 0.08	0.02 ± 0.01	7.8 ± 13.5	4.4 ± 8.5
P10	7.5 ± 0.8	0.00 ± 0.01	7.8 ± 1.1	8.7 ± 1.7	164 ± 27	0.75 ± 0.12	0.15 ± 0.00	47.1 ± 11.5	48.5 ± 7.7
P11	6.1 ± 0.7	0.07 ± 0.01	2.6 ± 0.4	4.2 ± 1.7	143 ± 58	0.78 ± 0.11	0.11 ± 0.03	57.5 ± 5.1	58.9 ± 3.2
P12	8.1 ± 0.7	0.06 ± 0.03	5.3 ± 1.0	6.1 ± 1.1	175 ± 3	0.80 ± 0.05	0.06 ± 0.03	5.7 ± 10.3	7.0 ± 10.6
Mean ± S*D*	9.1 ± 1.2	0.05 ± 0.02	4.8 ± 0.7	16.0 ± 7.6	118 ± 48	0.76 ± 0.14	0.09 ± 0.03	32.6 ± 12.2	31.1 ± 10.1
*R* ^2^	0.985	0.993	0.975	0.948	0.987	0.825	0.823	0.382	0.451

Linear regression analyses were used to assess how closely the recovered parameter values matched the parameter values derived from the bootstrapping procedure. The variance explained (*R*^2^) by the linear regression analyses ranged between 83 and 99% for the square frame. For the squircles, the explained variances were 38% (squircle 1) and 45% (squircle 2) for the gain factors (see Table 2 for a complete list).

## Discussion

In the current study, we manipulated frame form to investigate the impact of panoramic uncertainty on the vertical perception of a visually presented line. Orientation of the frame was found to (cyclically) bias the subjective visual vertical (SVV), and tilted frames were associated with larger variance in the responses, both indicative of a standard RFE. Furthermore, we replicated earlier findings on the effect of head tilt, demonstrating that roll-tilt of the head leads to a larger RFE. In addition to these replications, we found that rounder frame forms – which increase the uncertainty about the panoramic orientation – diminish the RFE. The modulation of the RFE by frame form was gradual in the sense that the intermediate steps from circle to square led step-by-step to a larger RFE.

With a squared frame, a clear RFE was observed, both in the bias and in the variability of the responses. When the frame was tilted leftward or rightward, the point of subjective equality was shifted, respectively, to the left or to the right, replicating many previous studies on the RFE (Witkin and Asch, 1948; see for a review: Medendorp et al., 2018). Responses to tilted frames were more variable than responses to the upright frame, in line with previous findings (Alberts et al., 2016; Niehof et al., 2019). Both effects, a bias and increased variance, were predicted based on the optimal Bayesian multisensory integration (MSI) account, which suggests that the prior experience of the observer that lines often match the cardinal axes will pull the vertical judgment toward the orientation of peripheral lines, and frame rotation leads to larger visual context uncertainty. Furthermore, congruent with optimal Bayesian MSI and in line with previous findings (DiLorenzo and Rock, 1982; Corbett and Enns, 2006; Vingerhoets et al., 2009; Alberts et al., 2016; De Winkel et al., 2021), the current study found a stronger RFE when participants had their head tilted by 30°compared to when they held their head upright.

Besides the classic SVV manipulations of varying the frame and head orientation, we manipulated frame form. The role of frame form on the RFE has historically been studied from the perspective of holistic processing in which different visual features could lead to the same gestalt (Koffka, 1935) and hence potentially to a similar RFE. The idea of the frame as a unitary stimulus, in terms of a gestalt, was reflected in the major axes hypothesis (Beh et al., 1971). According to this hypothesis, a major frame axis was defined as a line intersecting with the center of the frame which splits the frame into two symmetrical parts. The frame was thought to pull the vertical judgment of the rod by means of the major axis that was closest to the gravitational line. The major axes hypothesis was studied using triangular and hexagonal frames (Beh and Wenderoth, 1972). However, the idea of the frame functioning as a unitary stimulus was later abandoned as illusory shapes did not necessarily evoke an RFE (Ebenholtz, 1985), and independent lines (Li and Matin, 2005a, b; Vingerhoets et al., 2009) could function as visual context as well. As such, a frame is a specific instantiation of a set of peripheral lines that provide panoramic information about orientation. Frame form can thus affect the RFE not necessarily as a unitary stimulus, but through the degree to which its components have a clear orientation that can be mapped onto the cardinal axes (van der Schaaf and van Hateren, 1996; Coppola et al., 1998; Girshick et al., 2011).

Here, the employed frame form manipulation functioned as a means to alter the uncertainty of the orientation cue provided by the visual context. Reasoning from a Bayesian MSI account, a more uncertain visual cue should be assigned less weight in the head-in-space estimate and hence lead to a smaller impact on the visual context. This was indeed found. First, the results showed that increasing roundness reduced the RFE. Second, the modeling demonstrated that the rounder frame forms led to a larger panoramic uncertainty, as the gain factor was larger than one for both squircles. This indicates that the variability parameters belonging to the visual context were larger for the squircular frames compared to the squared frame. Indeed, for 9 of the 12 participants, the gain factor was higher for the rounder squircle compared to the more square-like squircle. In close connection, the study by Alberts et al. (2016) demonstrated that increased viewing distance could be modeled as an increase in visual context uncertainty through ramping up the gain factor. However, the reduced RFE for larger viewing distances that they found could be the result of uncertainty about the frame orientation, the rod orientation, or both. To address part of this issue, Pomante et al. (2019) showed that manipulating the orientation uncertainty of the central stimulus – ellipses with various eccentricities were used instead of rods – does not affect the bias in an ellipse-in-frame task, indicating that the central stimulus does not interact with the frame in global visual processing. These findings suggested that the result from Alberts et al. (2016) was probably indeed the result of increased uncertainty about the frame orientation and not about the rod. The current empirical and model findings provide further support that uncertainty about the orientation of the visual context impacts the perception of the earth vertical.

In terms of the model fits, the present model fitted the data better than a model-free description based on psychometric fits (ΔBIC across all subjects, Table 1). The model fit could likely be improved by measuring a larger range of rod orientations. Figure 2 indicates that the currently used range of rod orientations may have been too restricted. In earlier work using the same model, the same (−7 to 7°; Alberts et al., 2016), but also larger ranges have been utilized (−12 to 12°, Alberts et al., 2019; −15 to 15°, Alberts et al., 2018). Here, we opted for a restricted range to allow us to measure all four frame forms under a specific head orientation within one measurement session, accepting the limitations that come with such a restricted range.

A large part of the current study forms a replication of earlier work. Applying the same model to data collected under the same conditions led to parameter values within the same range as the earlier studies (Clemens et al., 2011; Alberts et al., 2016, 2018, 2019), emphasizing the robustness of the model, and replicability of the observed effects. The condition of interest, such as the manipulation of panoramic reliability with the use of squircular frames, was captured in the model by the gain parameter. While Alberts et al. (2016) reported an average gain of 1.31, we reported an average gain of 32.6 and 31.1, respectively, for squircles 1 and 2. These higher gain factors indicate that the uncertainty about the orientation of the visual context can more effectively be altered by changing the roundness of the frame form than by increasing the viewing distance. Large individual differences were observed in the gain factors, which may be a direct result of the large individual differences in σ*_*vert*_* and σ*_*hor*_*, which accounted for the reliability of the vertical and horizontal context information, respectively. Alberts et al. (2016) also observed large individual differences in these measures, with σ*_*vert*_* ranging between 1.8 and 10.2° and σ*_*hor*_* ranging between 30.2 and 104.6°. It could be argued that some individuals are more sensitive to visual context than others, in line with findings going back even to the early work of Witkin and Asch (1948). Indeed, older people, whose vestibular system is less sensitive than that of younger people, have been found to rely more strongly on a visual context in the rod-and-frame task (Alberts et al., 2019), which led to higher in σ*_*vert*_* and σ*_*hor*_* values in the model. Future research could reveal whether the impact of turning a square into a squircle is larger for the elderly or other populations experiencing vestibular loss.

The degree to which a sensory signal weighs into the final percept depends on its reliability, and on the reference frame of the task (Clemens et al., 2011, see also De Winkel et al., 2021). The maximum bias induced by the visual frame was 9.8°, comparable to the maximum bias observed by De Winkel et al. (2021). However, there were substantial individual differences [the smallest bias we observed was 1.9° (*SD* = 2.0°)], which are also reflected in the individual differences in sensory weights (see also Alberts et al., 2018, 2019). The weight of the prior on head orientation ranged between 0.03 and 0.38 (Mean = 0.20), the visual weight ranged between 0 and 0.47 (Mean = 0.24), and the vestibular weight ranged between 0.32 and 0.74 (Mean = 0.57). These values are very comparable to the weights found by Alberts et al. (2018, 2019). With increasing roundness of the frame, the prior on head orientation gained slightly in weight, the vestibular weight increased and the visual weight decreased. Although the size of these changes varied between participants, the pattern was found in every individual.

Our findings provide further evidence for the notion that vertical perception is the result of Bayes-optimal MSI, in which weights are assigned to each cue relative to its reliability. In the real world, the visual context often contains many lines and polarity cues, and hence as a next step, we propose to investigate how visual context reliability as assessed by the model relates to the orientation of a multitude of line segments in the periphery. If the context solely consists of randomly oriented lines, it no longer can function as head orientation, and hence a verticality cue, and thus will have a reliability of zero. If the context purely consists of vertical lines, its reliability as a verticality cue is maximal but will decrease if more randomly oriented lines are intermixed.

To conclude, the current study demonstrated that panoramic uncertainty, manipulated through changes in frame form, altered the RFE. The RFE bias was stronger for a fairly square-like squircle compared to a rounder squircle, and a regular square had a stronger impact than both squircles. The weaker the orientation cues conveyed by visually presented abstract frame form, the smaller the impact of this visual context on vertical judgments of a visual line, congruent with the Bayesian ideal observer model.

## Data Availability Statement

The datasets presented in this study can be found in an online repository. The name of the repository and accession number can be found below: https://doi.org/10.34973/kbzc-ng08.

## Ethics Statement

The studies involving human participants were reviewed and approved by Ethics Committee Social Science, Radboud University Nijmegen. The participants provided their written informed consent to participate in this study.

## Author Contributions

JCS and WPM: conceptualization, methodology, and modeling. JCS: data collection, analyses, and writing – original draft. WPM: writing – review and editing. Both authors contributed to the article and approved the submitted version.

## Conflict of Interest

The authors declare that the research was conducted in the absence of any commercial or financial relationships that could be construed as a potential conflict of interest.

## Publisher’s Note

All claims expressed in this article are solely those of the authors and do not necessarily represent those of their affiliated organizations, or those of the publisher, the editors and the reviewers. Any product that may be evaluated in this article, or claim that may be made by its manufacturer, is not guaranteed or endorsed by the publisher.
